# Complete genome of *Nitrosospira briensis* C-128, an ammonia-oxidizing bacterium from agricultural soil

**DOI:** 10.1186/s40793-016-0168-4

**Published:** 2016-07-28

**Authors:** Marlen C. Rice, Jeanette M. Norton, Frederica Valois, Annette Bollmann, Peter J. Bottomley, Martin G. Klotz, Hendrikus J. Laanbroek, Yuichi Suwa, Lisa Y. Stein, Luis Sayavedra-Soto, Tanja Woyke, Nicole Shapiro, Lynne A. Goodwin, Marcel Huntemann, Alicia Clum, Manoj Pillay, Nikos Kyrpides, Neha Varghese, Natalia Mikhailova, Victor Markowitz, Krishna Palaniappan, Natalia Ivanova, Dimitrios Stamatis, T. B. K. Reddy, Chew Yee Ngan, Chris Daum

**Affiliations:** 1 Utah State University, Logan, UT USA; 2 Woods Hole Oceanographic Institution, Woods Hole, MA USA; 3 Miami University, Oxford, OH USA; 4 Oregon State University, Corvallis, OR USA; 5 Queens College in The City University of New York, Flushing, NY USA; 6 The Institute of Marine Microbes and Ecospheres, Xiamen University, Xiamen, China; 7 Netherlands Institute of Ecology, Wageningen, The Netherlands; 8 Utrecht University, Utrecht, The Netherlands; 9 Chuo University, Tokyo, Japan; 10 University of Alberta, Edmonton, AB Canada; 11 DOE Joint Genome Institute, Walnut Creek, CA USA; 12 Los Alamos National Laboratory, Bioscience Division, Los Alamos, NM USA

**Keywords:** *Nitrosospira*, Ammonia-oxidizing bacteria, Nitrification, Agricultural soil, Ammonia monooxygenase, Nitrous oxide, Chemolithotroph

## Abstract

*Nitrosospira briensis* C-128 is an ammonia-oxidizing bacterium isolated from an acid agricultural soil. *N. briensis* C-128 was sequenced with PacBio RS technologies at the DOE-Joint Genome Institute through their Community Science Program (2010). The high-quality finished genome contains one chromosome of 3.21 Mb and no plasmids. We identified 3073 gene models, 3018 of which are protein coding. The two-way average nucleotide identity between the chromosomes of *Nitrosospira multiformis* ATCC 25196 and *Nitrosospira briensis* C-128 was found to be 77.2 %. Multiple copies of modules encoding chemolithotrophic metabolism were identified in their genomic context. The gene inventory supports chemolithotrophic metabolism with implications for function in soil environments.

## Introduction

The first step in the aerobic nitrification process is the oxidation of ammonia to nitrite, mediated mainly by AOB or AOA in soil environments. The most numerous AOB isolated or detected by non-cultural methods in aerobic agricultural surface soils are consistently members of the *Nitrosospira* genus [1]. *Nitrosospira briensis* C-128 [2] is a chemolithoautotrophic ammonia-oxidizing betaproteobacterium (order *Nitrosomonadales*, family *Nitrosomonadaceae**,* genus *Nitrosospira* [3–9]) isolated from a fertilized soil under cultivation for blueberry in Falmouth, Massachusetts, USA in 1971. The genome of *Nitrosospira briensis* C-128 is the third genome sequence from the genus *Nitrosospira* [8–10] to be published [11–13] and thus provides an important comparison among *Nitrosospira**.* This report includes a summary of the genome sequence and selected features for *Nitrosospira briensis* C-128 and results are publically available in GenBank accession CP012371.

## Organism information

### Classification and features

**Fig. 1:**
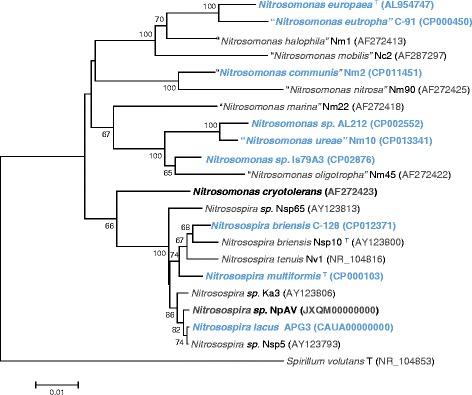
The phylogenetic tree highlighting the position of *Nitrosospira briensis* C-128 relative to other *Nitrosomonadaceae* [48] *and Spirillum volutans* (outgroup). The tree was inferred from 1417 aligned characters of the 16S rRNA gene sequence by the neighbour-joining method [49] using the sotware MEGA [50]. Support values (%) at branch points are from 1000 NJ bootstrap replicates and shown only for values exceeding 60 %. GenBank references are for genomes (full-length 16S rRNA gene extracted) or are the near full-length 16S rRNA sequences [51]. Bold denotes a genome sequence available (NCBI or GOLD), whereas bold blue denotes the published genomes: *Nitrosomonas europaea* [52], *“Nitrosomonas eutropha”* [53], “*Nitrosomonas communis”* [54], *Nitrosomonas sp.* AL212 [55], *“Nitrosomonas ureae”* [56], *Nitrosomonas sp.* Is79A3 [57], *Nitrosospira briensis* C-128 (this study), *Nitrosospira lacus* APG3 [11] and *Nitrosospira multiformis* [12]

**Fig. 2:**
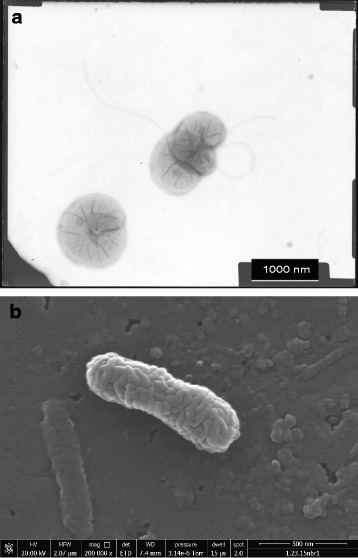
Electron micrographs of *N. briensis*. A) TEM prepared by negative staining as previously described [14, 15]. Scale is 1000 nm. B) SEM of *Nitrosospira briensis* C-128. Glass coverslips were placed in a growing culture for approximately one month, removed and then fixed with 2 % glutaraldehyde in 0.1 % HEPES buffer overnight. The samples were subjected to alcohol series dehydration (50-100 % ethanol) and then chemically dried using hexamethyldisilazane. The image shows presumptive invaginations of the membranes of the cell. Scale is 500 nm

*Nitrosospira briensis* was described by Winogradsky and & Winogradsky in 1933 [8] as an ammonia-oxidizing bacterium isolated from soil. The genus name, *Nitrosospira**,* is derived from two Latin roots: nitrosus, meaning nitrous, and spira, indicating spiral. The species name *briensis*, refers to the original isolation location near Brie, France. The culture described by Winogradsky & Winogradsky [8] was not maintained and reisolation of a replacement strain was reported by Watson in 1971 [14]. At approximately the same time, *N. briensis* strain C-128 was isolated by enrichment culturing [15] from a surface soil sample (pH 6.2) collected from a fertilized blueberry patch in East Falmouth, Massachusetts in 1971 (Frederica Valois). In 1993, the genus *Nitrosospira* was emended to include the former genera of *Nitrosovibrio* and *Nitrosolobus* [9] based on the high identities of the 16S rRNA gene sequences. *Nitrosospira briensis* was designated the type species for the genus with strain C-76 as the type strain (also known as strain Nsp10 [16]^1^). The full-length 16S rRNA gene sequence of *N. briensis* C-128 is 99 % identical to the *N. briensis* strain C-76/Nsp10 sequence (Fig. 1). The culture of *N. briensis* strain C-128 was received in the Norton laboratory from F. Valois (Woods Hole Oceanographic Institution) in 1995. *Nitrosospira briensis* C-128 is presently maintained in a culture collection at WHOI and may be obtained upon request from J.M. Norton. Classification and general features of *Nitrosospira briensis* C-128 are provided as *Minimum Information about the Genome Sequence* (MIGS) in Table 1. Electron micrographs of the pure culture organism are shown in Fig. 2 revealing the tight spirals visible with TEM negative staining and the convoluted surface of this *Nitrosospira* as revealed by SEM.

**Table 1:** Classification and general features of *Nitrosospira briensis* C-128 [42, 43]

MIGS ID	Property	Term	Evidence code^a^
	Current classification	Domain *Bacteria*	TAS [44]
		Phylum *Proteobacteria*	TAS [45]
		Class *Betaproteobacteria*	TAS [7, 46]
		Order *Nitrosomonadales*	TAS [5, 46]
		Family *Nitrosomonadaceae*	TAS [4, 46]
		Genus *Nitrosospira*	TAS [6, 8]
		Species *Nitrosospira briensis*	TAS [6, 8]
		Strain C-128	IDA
	Gram stain	negative	TAS [14]
	Cell shape	Spiral/vibrioid	IDA
	Motility	motile	TAS [14]
	Sporulation	Non-sporulating	TAS [14]
	Temperature range	15–30 °C	TAS [14]
	Optimum temperature	25–28 °C	TAS [14]
	pH range; Optimum	6.0–8.2;7.0	TAS [14]
	Carbon source	carbon dioxide; carbonate	TAS [14]
	Energy source	ammonia oxidation	TAS [14]
	Energy metabolism	chemolithotroph	TAS [14]
MIGS-6	Habitat	soil (acid)	IDA
MIGS-6.3	Salinity	Non-halophile	TAS [14]
MIGS-22	Oxygen requirement	Aerobic	TAS [14]
MIGS-23	Isolation and growth conditions	Isolation after enrichment on inorganic ammonium salts medium	TAS [14]
MIGS-15	Biotic relationship	Free living	NAS
MIGS-14	Pathogenicity	Non-pathogen	NAS
	Biosafety level	1	NAS
MIGS-4	Geographic location	East Falmouth, MA, USA	NAS
MIGS-4.1	Latitude	41°35′38″ N	NAS
MIGS-4.2	Longitude	70°34′20″ W	NAS
MIGS-4.3	Depth	surface soil	NAS
MIGS-4.4	Altitude	6 m	NAS
MIGS-5	Sample collection	1971 Feb 18	NAS

^a^Evidence codes – *IDA* Inferred from Direct Assay (first time in publication), *TAS* Traceable Author Statement (i.e. a direct report exists in the literature), *NAS* Non-traceable Author Statement (i.e. not directly observed for living, isolated sample, but based on a generally accepted property for the species, or anecdotal evidence). These evidence codes are from the Gene Ontology project [47]

## Genome sequencing information

### Genome project history

*Nitrosospira briensis* C-128 was chosen for sequencing through the Community Science Program (2010) of the DOE Joint Genome Institute as an important representative of the AOB to improve the scope and quality of intra- and inter-generic comparisons in the *Nitrosomonadales*. The chemolithotrophic metabolism of the AOB, the pathways for production of nitrous oxide and urea metabolism were additional motivating interests in sequencing this genome. Sequencing, finishing, and annotation were accomplished by JGI. The genome sequence has been deposited in the Genome OnLine Database [17] and is part of the NCBI Reference Sequence Collection [18]. A summary of the project information is found in Table 2.

**Table 2:** Genome sequencing project information

MIGS ID	Property	Term
MIGS 31	Finishing quality	Finished
MIGS-28	Libraries used	One library, PacBio SMRTbell Library
MIGS 29	Sequencing platforms	PacBio RS
MIGS 31.2	Fold coverage	176X
MIGS 30	Assemblers	HGAP v. 2.2.0.p1 [23]
MIGS 32	Gene calling method	Prodigal, GenePRIMP
	Locus Tag	F822
	Genbank ID	CP012371.1
	GenBank Release Date	14-Aug-2015
	GOLD ID	Gp0006506
	BioProject ID	PRNJA183056
MIGS 13	Source Material Identifier	*Nitrosospira briensis* C-128 WHOI
	Project relevance	Environmental, Biogeochemical cycling of nitrogen, Biotechnological

### Growth conditions and genomic DNA preparation

*Nitrosospira briensis* C-128 was grown in a 25 mM ammonium medium pH 7 containing mineral salts and phenol red at 28 °C in 100 ml of media in 500 ml flasks as described previously [19]. The pH was adjusted to neutral using 0.5 M KHCO_3_ as needed during growth. Early stationary phase cultures were checked at harvest for heterotrophic contamination by plating 0.1 mL on ¼ strength nutrient agar plates and incubating for two weeks. Cells were harvested from four 100 mL cultures by centrifugation (13,000 RCF for 30 min). Bacterial genomic DNA (gDNA) was isolated using the CTAB protocol recommended by JGI [20]. Size and quality of the gDNA was assessed via gel electrophoresis and amplification of the V4 region of the 16S rRNA gene using universal primers [21] followed by sequencing at the Center for Integrative Biosystems, USU on the ABI PRISM™ 3730 DNA Analyzer using BigDye terminator chemistry. The gDNA was of the expected size (greater than 23 kbp) and no contaminating organisms were detected by partial 16S rRNA gene sequencing of 10 replicate reactions or by plating. Approximately 20 μg of DNA was submitted to JGI for sequencing.

### Genome sequencing and assembly

The genomic DNA of *Nitrosospira briensis* C-128 was sequenced at the DOE JGI using the Pacific Biosciences (PacBio) sequencing technology [22]. All general aspects of sample handling, library construction and sequencing followed JGI isolate sequencing protocols. A PacBio SMRTbell™ library was constructed and sequenced on the PacBio RS platform, which generated 148,206 reads totaling 519.8Mbp. Raw reads were assembled using HGAP v. 2.2.0.p1 [23]. The final draft assembly contained one contig in one scaffold, totaling 3.2 Mbp in size. The input read coverage was 176.1×. An earlier version of the genome was sequenced using the Illumina Hi-Seq 2000 platform. However, this earlier sequence assembly JHVX00000000.1 remained in 31 scaffolds (sequences JHVX01000001.1-JHVX01000031.1) with the nearly identical repeats of several key catabolic gene clusters remaining unresolved. Previously, genome closure for *Nitrosospira* [12] was achieved only after extensive directed finishing to correctly assemble long nearly identical repeats of gene clusters encoding key catabolic modules including ammonia monooxygenase (*amo*) for the activation of substrate and hydroxylamine dehydrogense (*haoA*) and heme-cytochrome *c* proteins (*cycAB*) for the extraction of electrons and their delivery to the quinone pool in the membrane [24]. The long read capability of the PacBio platform and our depth of coverage enabled sufficient discrimination of repeats to assemble across multiple nearly identical regions into a single contig representing the chromosome of the bacterium. For predicted genes outside of gaps and repeat regions the PacBio and the Illumina predicted genes were 100 % identical. Therefore, we did not combine the Illumina Hi-Seq data with the PacBio data for the complete genome sequence CP012371 reported here.

### Genome annotation

Genes were identified using Prodigal [25], as part of the JGI’s Microbial annotation pipeline followed by a round of manual curation using GenePRIMP [26]. The predicted CDSs were translated and used to search the NCBI nonredundant database, UniProt, TIGRFam, Pfam, KEGG, COG, and InterPro databases. Transfer RNA genes were identified using the tRNAScanSE tool [27]. Ribosomal RNA genes were found by searches against models of the ribososmal RNA genes built from SILVA [28]. Other non-coding RNAs were found using INFERNAL [29]. Further gene prediction and manual curation was performed within the Integrated Microbial Genomes (IMG) platform [30] developed at JGI.

### Genome properties

**Fig. 3:**
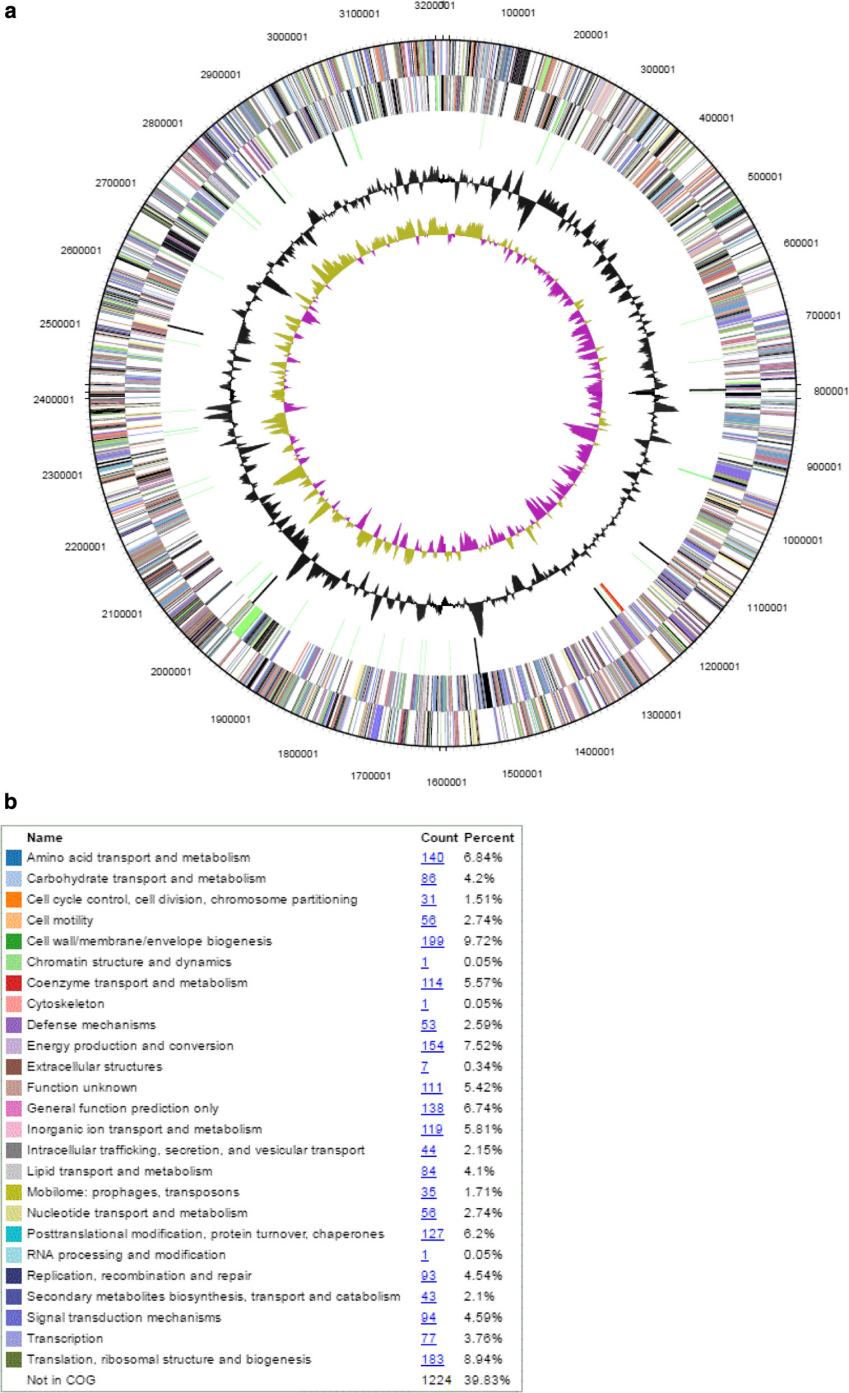
**a** Graphical map of the genome. From the outside to the center: genes on forward stand and Genes on reverse strand (color by COG categories see legend), RNA genes (tRNAs green, rRNAs red, other RNAs black), GC content, GC skew. **b** Legend for COG category colors

The genome of *Nitrosospira briensis* C-128 contains 3,210,113-bp in one chromosome with a GC content of 53.25 % and no plasmids (Fig. 3). The genome contains one complete ribosomal RNA operon similar to other AOB [3]. Coding bases (2,758,471) comprised 85.93 % of the total. We identified 3018 protein encoding genes, 55 RNA genes and 130 pseudogenes. For the identified genes, 74.23 % had a function prediction associated with them. The two-way average nucleotide identity [31] between the chromosomes of *Nitrosospira multiformis* ATCC 25196 [9, 32, 33] and *Nitrosospira briensis* C-128 was found to be 77.2 % confirming species delineation [34]. The genome statistics are summarized in Table 3 and genes associated with COG functional categories are summarized in Table 4.

**Table 3:** Genome statistics

Attribute	Value	% of Total
Genome size (bp)	3,210,113	100.00
DNA coding (bp)	2,758,471	85.93
DNA G + C (bp)	1,709,486	53.25
DNA scaffolds	1	100.00
Total genes	3073	100.00
Protein coding genes	3018	98.2
RNA genes	55	1.79
Pseudo genes	130	4.23
Genes with internal clusters	394	12.82
Genes with function prediction	2232	72.63
Genes assigned to COGs	1849	60.17
Genes with Pfam domains	2303	74.94
Genes with signal peptides	290	9.44
Genes with transmembrane helices	741	24.11
CRISPR repeats	1	0.02

**Table 4:** Number of genes associated with general COG functional categories

Code	Value	%age	Description
J	149	7.32	Translation, ribosomal structure and biogenesis
A	1	0.05	RNA processing and modification
K	82	4.03	Transcription
L	122	6.00	Replication, recombination and repair
B	1	0.05	Chromatin structure and dynamics
D	25	1.23	Cell cycle control, Cell division, chromosome partitioning
V	24	1.18	Defense mechanisms
T	75	3.69	Signal transduction mechanisms
M	167	8.21	Cell wall/membrane biogenesis
N	53	2.60	Cell motility
U	67	3.29	Intracellular trafficking and secretion
O	114	5.60	Posttranslational modification, protein turnover, chaperones
C	153	7.52	Energy production and conversion
G	88	4.32	Carbohydrate transport and metabolism
E	140	6.88	Amino acid transport and metabolism
F	54	2.65	Nucleotide transport and metabolism
H	99	4.86	Coenzyme transport and metabolism
I	73	3.59	Lipid transport and metabolism
P	106	5.21	Inorganic ion transport and metabolism
Q	57	2.80	Secondary metabolites biosynthesis, transport and catabolism
R	202	9.93	General function prediction only
S	183	8.99	Function unknown
-	1224	39.83	Not in COGs

## Insights from the genome sequence

### Selected functional inventory in the complete genome sequence

*Nitrosospira briensis* C-128 contains complete “*amo”* and “*hao”* gene clusters in three nearly identical copies on the chromosome. The full-length *amoCABEDcopCD* gene cluster is repeated twice (F822_1680-1686, & 2228–2234) while the third cluster contains only the three structural “*amo*” genes, *amoCAB* (F822_0880-0878). As in most other betaproteobacterial AOB genomes, the *N. briensis* C-128 genome contains three additional *amoC* singleton genes (F822_0485, 1530, & 2742). The “hydroxylamine-ubiquinone redox module” (HURM) [24] is encoded by the *haoAB-cycAB* gene cluster, which occurs three times (F822_0640-0643, 0873–0876, 1808–1811) in the genome sequence. The *N. briensis* C-128 genome also encodes nitrosocyanin (*ncyA*; F822_2886), a protein unique to ammonia-oxidizing bacteria, which possibly functions in the regulation of electron transfer [35]. A urease operon containing α, β, & γ subunit-encoding genes as well as genes encoding accessory proteins E, F, G, & H (F822_0450-0456) is preceded by a urea transporter gene (*utp*; F822_0449). Genes encoding alternative catabolic inventory such as hydrogenase were not identified. The *N. briensis* C-128 genome contains a single gene cluster encoding the Calvin-Benson-Bassham cycle for carbon assimilation including the carboxylation reaction, which is encoded by a single-copy *cbb* operon in the Form 1C (red-like) subgroup (F822_1009-1012) with > 90 % identity with homologous genes in *Nitrosospira multiformis* [36] and *Nitrosospira**sp.* 40KI [37].

Genes encoding inventory implicated in nitrogen oxide metabolism and /or nitrosative stress [38] include those for copper nitrite reductase (*nirK*, singleton F822_2604) and a possible quinol nitric oxide reductase (qNOR) encoding gene (F822_0115). Similar to arrangements in many AOB genomes, a gene cluster (*norSY-senC-orf1*) (F822_ 1803–1806) encoding nitric oxide reductase heme-copper oxidase (sNOR) was found upstream of a nitrite transporter gene (F822_1807) and one of the three *haoAB-cycAB* clusters. However, the *norCBQD* cluster encoding cytochrome C nitric oxide reductase (cNOR) was not found. The genes encoding precursors of cytochromes *c*’-beta (*cytS*) and P-460 (*cytL*) were not detected in the C-128 genome sequence. The gene of NO-responsive regulator (*nnrS*) was present albeit truncated.

CRISPR/Cas System *Nitrosospira briensis* C-128 contains a CRISPR/Cas system located at F822_1846-1851 suggestive of phage interactions [39]. The CRISPR-associated (CAS) proteins belong to the subtype 1-F (*Yersinia pestis* type) [40]. The CRISPR contains 11 spacers each with 32 bp. No matches between these spacers and protospacers in viral genomes were detected in the NCBI non-redundant database. The direct repeat sequence in the CRISPR is 28 bp: TTTCTGAGCTGCCTATGCGGCAGTGAAC. As soil viral metagenomes become better characterized, associations between viral protospacers and the spacers found in *N. briensis**’* CRISPR may help to identify possible phage types of *N. briensis*.

## Conclusions

*Nitrosospira briensis* C-128 has a suite of genes enabling it to survive in soil environments as a chemolithoautotroph. The completion of several genomes in the *Nitrosospira* genus will facilitate a comprehensive analysis of the genetic toolkit that enables these AOB to co-inhabit the terrestrial niche. Further experiments elucidating gene function, especially those involved in the metabolism of nitrogen oxides and related to nitrosative stress [41], will increase the relevance of the completed genome of *Nitrosospira briensis* C-128. The evolutionary relationships in the genera of the *Nitrosomonadaceae* are currently under reconsideration.

## Abbreviations

AOA: Ammonia-oxidizing archaea
AOB: Ammonia-oxidizing bacteria
WHOI: Woods Hole Oceanographic Institution

## Footnotes

1 Editor’s note – Readers are advised that the published record regarding the type strain and a proposed neotype strain of *Nitrosospira briensis* is problematic. Although the Approved Lists of Bacterial Names (I*nt J Syst Bacteriol* 1980; **30**:225) list the type as “no culture available”, Koops and Harms subsequently published on strain Nsp 10 as the equivalent to ATCC 25971 (Arch Microbiol 1985; **141**:214–218)

## References

[1] Norton JM, Schepers JS, Raun WR (2008). Nitrification in Agricultural Soils. Nitrogen in Agricultural Systems.

[2] Exemplar Abstract for *Nitrosospira briensis* C-128. NamesforLife, LLC. Retrieved May 4, 2016. http://doi.org/10.1601/ex.18586

[3] Prosser JI, Head IM, Stein L, Rosenberg E, DeLong E, Lory S, Stackebrandt E, Thompson F (2014). The family *Nitrosomonadaceae*. The prokaryotes.

[4] Garrity G, Bell J, Lilburn T, Family I, Garrity G, Brenner D, Krieg N, Staley J (2005). *Nitrosomonadaceae* fam. nov. Bergey’s manual of systematic bacteriology volume 2 part C.

[5] Garrity G, Bell J, Lilburn T, Order V, Garrity G, Brenner D, Krieg N, Staley J (2005). *Nitrosomonadales* ord. Bergey’s manual of systematic bacteriology volume 2 part C.

[6] Skerman V, McGowan V, Sneath P, Moore W, Moore LV (1980). Approved lists of bacterial names. Int J Syst Bacteriol.

[7] Garrity G, Bell J, Lilburn T, Class II, Garrity G, Brenner D, Krieg N, Staley J (2005). *Betaproteobacteria* class. nov. Bergey’s manual of systematic bacteriology. Volume 2 part C.

[8] Winogradsky S, Winogradsky H (1933). Etudes sur la microbiologie du sol. VII. Nouvelles recherches sur les organismes de la nitrification. Ann Inst Pasteur (Paris).

[9] Head IM, Hiorns WD, Embley TM, McCarthy AJ, Saunders JR (1993). The phylogeny of autotrophic ammonia -oxidizing bacteria as determined by analysis of 16S ribosomal -RNA sequences. J Gen Microbiol.

[10] Name Abstract for *Nitrosospira*. NamesforLife, LLC. Retrieved May 5, 2016. http://doi.org/10.1601/nm.2005.

[11] Garcia JC, Urakawa H, Le VQ, Stein LY, Klotz MG, Nielsen JL (2013). Draft genome sequence of *Nitrosospira sp.* strain APG3, a psychrotolerant ammonia-oxidizing bacterium isolated from sandy lake sediment. Genome Announc.

[12] Norton JM, Klotz MG, Stein LY, Arp DJ, Bottomley PJ, Chain PSG, Hauser LJ, Land ML, Larimer FW, Shin MW (2008). Complete genome sequence of *Nitrosospira multiformis*, an ammonia-oxidizing bacterium from the soil environment. Appl Environ Microbiol.

[13] Urakawa H, Garcia JC, Nielsen JL, Le VQ, Kozlowski JA, Stein LY, Lim CK, Pommerening-Röser A, Martens-Habbena W, Stahl DA (2015). *Nitrosospira lacus* sp. nov., a psychrotolerant, ammonia-oxidizing bacterium from sandy lake sediment. Int J Syst Evol Microbiol.

[14] Watson SW (1971). Reisolation of *Nitrosospira briensis* S. Winogradsky and H. Winogradsky 1933. Arch Mikrobiol.

[15] Watson S, Valois F, Waterbury J, Starr MP, Stolp H, Schlegel HG (1981). The family *Nitrobacteraceae*. Prokaryotes: a handbooks on habits, isolation, and identification of bacteria.

[16] Watson SW (1971). Taxonomic considerations of the family *Nitrobacteraceae* Buchanan. Int J Syst Bacteriol.

[17] Reddy TB, Thomas AD, Stamatis D, Bertsch J, Isbandi M, Jansson J, Mallajosyula J, Pagani I, Lobos EA, Kyrpides NC (2015). The Genomes OnLine Database (GOLD) v. 5: a metadata management system based on a four level (meta)genome project classification. Nucleic Acids Res.

[18] Tatusova T, Ciufo S, Fedorov B, O’Neill K, Tolstoy I (2014). RefSeq microbial genomes database: new representation and annotation strategy. Nucleic Acids Res.

[19] Norton JM, Alzerreca JJ, Suwa Y, Klotz MG (2002). Diversity of ammonia monooxygenase operon in autotrophic ammonia-oxidizing bacteria. Arch Microbiol.

[20] Feil WS, Feil H, Copeland A. Bacterial DNA Isolation, CTAB Protocol. DOE Joint Genome Institute [http://1ofdmq2n8tc36m6i46scovo2e.wpengine.netdna-cdn.com/wp-content/uploads/2014/02/JGI-Bacterial-DNA-isolation-CTAB-Protocol-2012.pdf]. Accessed 19 Apr 2016.

[21] He ZL, Xu MY, Deng Y, Kang SH, Kellogg L, Wu LY, Van Nostrand JD, Hobbie SE, Reich PB, Zhou JZ (2010). Metagenomic analysis reveals a marked divergence in the structure of belowground microbial communities at elevated CO2. Ecol Lett.

[22] Eid J, Fehr A, Gray J, Luong K, Lyle J, Otto G, Peluso P, Rank D, Baybayan P, Bettman B (2009). Real-time DNA sequencing from single polymerase molecules. Science.

[23] Chin CS, Alexander DH, Marks P, Klammer AA, Drake J, Heiner C, Clum A, Copeland A, Huddleston J, Eichler EE (2013). Nonhybrid, finished microbial genome assemblies from long-read SMRT sequencing data. Nat Methods.

[24] Simon J, Klotz MG (2013). Diversity and evolution of bioenergetic systems involved in microbial nitrogen compound transformations. Biochim Biophys Acta (BBA)-Bioenergetics.

[25] Hyatt D, Chen G-L, LoCascio PF, Land ML, Larimer FW, Hauser LJ (2010). Prodigal: prokaryotic gene recognition and translation initiation site identification. BMC Bioinformatics.

[26] Pati A, Ivanova NN, Mikhailova N, Ovchinnikova G, Hooper SD, Lykidis A, Kyrpides NC (2010). GenePRIMP: a gene prediction improvement pipeline for prokaryotic genomes. Nat Methods.

[27] Lowe TM, Eddy SR (1997). tRNAscan-SE: a program for improved detection of transfer RNA genes in genomic sequence. Nucleic Acids Res.

[28] Pruesse E, Quast C, Knittel K, Fuchs BM, Ludwig W, Peplies J, Glöckner FO (2007). SILVA: a comprehensive online resource for quality checked and aligned ribosomal RNA sequence data compatible with ARB. Nucleic Acids Res.

[29] Nawrocki EP, Eddy SR (2013). Infernal 1.1: 100-fold faster RNA homology searches. Bioinformatics.

[30] Markowitz VM, Chen I-MA, Palaniappan K, Chu K, Szeto E, Pillay M, Ratner A, Huang J, Woyke T, Huntemann M. IMG 4 version of the integrated microbial genomes comparative analysis system. Nucleic Acids Res. 2013; gkt963 10.1093/nar/gkt963 PMC3965111 24165883

[31] Goris J, Konstantinidis KT, Klappenbach JA, Coenye T, Vandamme P, Tiedje JM (2007). DNA–DNA hybridization values and their relationship to whole-genome sequence similarities. Int J Syst Evol Microbiol.

[32] List_Editor. Validation List no. 54. Validation of the publication of new names and new combinations previously effectively published outside the IJSB. Int J Syst Bacteriol. 1995;45:619–620.

[33] Watson SW, Graham LB, Remsen CC, Valois FW (1971). A lobular, ammonia-oxidizing bacterium, *Nitrosolobus multiformis* nov. gen. nov. sp. Arch Mikrobiol.

[34] Konstantinidis KT, Ramette A, Tiedje JM (2006). The bacterial species definition in the genomic era. Phil Trans R Soc B.

[35] Stein LY, Campbell MA, Klotz MG (2013). Energy-mediated vs. ammonium-regulated gene expression in the obligate ammonia-oxidizing bacterium, *Nitrosococcus oceani*. Front Microbiol.

[36] Name Abstract for *Nitrosospira multiformis*. NamesforLife, LLC. Retrieved May 4, 2016. http://doi.org/10.1601/nm.2007.

[37] Utaker JB, Andersen K, Aakra A, Moen B, Nes IF (2002). Phylogeny and functional expression of ribulose 1,5-bisphosphate carboxylase/oxygenase from the autotrophic ammonia-oxidizing bacterium *Nitrosospira* sp. isolate 40KI. J Bacteriol.

[38] Stein LY, Klotz MG (2011). Surveying N_2_O-producing pathways in bacteria. Methods in enzymology: research on nitrification and related processes, Vol 486, Part A.

[39] Deveau H, Garneau JE, Moineau S (2010). CRISPR/Cas system and its role in phage-bacteria interactions. Annu Rev Microbiol.

[40] Makarova KS, Haft DH, Barrangou R, Brouns SJJ, Charpentier E, Horvath P, Moineau S, Mojica FJM, Wolf YI, Yakunin AF (2011). Evolution and classification of the CRISPR-Cas systems. Nat Rev Microbiol.

[41] Kozlowski JA, Price J, Stein LY (2014). Revision of N_2_O-producing pathways in the ammonia-oxidizing bacterium *Nitrosomonas europaea* ATCC 19718. Appl Environ Microbiol.

[42] Chain PSG, Grafham DV, Fulton RS, FitzGerald MG, Hostetler J, Muzny D, Ali J, Birren B, Bruce DC, Buhay C (2009). Genome project standards in a new era of sequencing. Science.

[43] Field D, Garrity G, Gray T, Morrison N, Selengut J, Sterk P, Tatusova T, Thomson N, Allen MJ, Angiuoli SV (2008). The minimum information about a genome sequence (MIGS) specification. Nat Biotechnol.

[44] Woese CR, Kandler O, Wheelis ML (1990). Towards a natural system of organisms: proposal for the domains Archaea, Bacteria, and Eucarya. Proc Natl Acad Sci U S A.

[45] Garrity G, Bell J, Lilburn T, Garrity G, Brenner D, Krieg N, Staley J (2005). Phylum XIV. *Proteobacteria* phyl. nov. Bergey’s manual of systematic bacteriology. Volume 2, Part B.

[46] List_Editor. Validation list No.107. List of new names and new combinations previously effectively, but not validly, published. Int J Syst Evol Microbiol. 2006;56(1):1–6. 10.1099/ijs.0.64188-0 16403855

[47] Ashburner M, Ball CA, Blake JA, Botstein D, Butler H, Cherry JM, Davis AP, Dolinski K, Dwight SS, Eppig JT (2000). Gene Ontology: tool for the unification of biology. Nat Genet.

[48] Name Abstract for *Nitrosomonadaceae*. NamesforLife, LLC. Retrieved May 5, 2016. http://doi.org/10.1601/nm.1991

[49] Saitou N, Nei M (1987). The neighbor-joining method: a new method for reconstructing phylogenetic trees. Mol Biol Evol.

[50] Tamura K, Stecher G, Peterson D, Filipski A, Kumar S (2013). MEGA6: molecular evolutionary genetics analysis version 6.0.. Mol Biol Evol.

[51] Koops HP, Bottcher B, Moller UC, Pommerening-Roser A, Stehr G (1991). Classification of eight new species of ammonia-oxidizing bacteria: *Nitrosomonas communis* sp nov., *Nitrosomonas ureae* sp nov., *Nitrosomonas aestuarii* sp nov., *Nitrosomonas marina* sp nov., *Nitrosomonas nitrosa* sp nov., *Nitrosomonas oligotropha* sp nov., *Nitrosomonas halophila* sp nov. J Gen Microbiol.

[52] Chain PSG, Lamerdin J, Larimer F, Regala W, Lao V, Land M, Hauser L, Hooper A, Klotz M, Norton J (2003). Complete genome sequence of the ammonia-oxidizing bacterium and obligate chemolithoautotroph *Nitrosomonas europaea*. J Bacteriol.

[53] Stein LY, Arp DJ, Berube PM, Chain PSG, Hauser L, Jetten MSM, Klotz MG, Larimer FW, Norton JM, Op den Camp HJ (2007). Whole-genome analysis of the ammonia-oxidzing bacterium, *Nitrosomonas eutropha* C91: implications for niche adaptation. Environ Microbiol.

[54] Kozlowski JA, Kits KD, Stein LY (2016). Genome sequence of *Nitrosomonas communis* strain Nm2, a mesophilic ammonia-oxidizing bacterium isolated from mediterranean soil. Genome Announc.

[55] Suwa Y, Norton JM, Bollmann A, Klotz MG, Stein LY, Laanbroek HJ, Arp DJ, Goodwin LA, Chertkov O, Held B (2011). Genome sequence of *Nitrosomonas sp*. Strain AL212, an ammonia-oxidizing bacterium sensitive to high levels of ammonia. J Bacteriol.

[56] Kozlowski JA, Kits KD, Stein LY (2016). Complete genome sequence of *Nitrosomonas ureae* strain Nm10, an Oligotrophic Group 6a Nitrosomonad. Genome Announc.

[57] Bollmann A, Sedlacek CJ, Norton JM, Laanbroek HJ, Suwa Y, Stein LY, Klotz MG, Arp DJ, Sayavedra-Soto L, Goodwin LA (2013). Complete genome sequence of *Nitrosomonas* sp. Is79 - an ammonia oxidizing bacterium adapted to low ammonium concentrations. Stand Genomic Sci.

